# Lockdown during the COVID-19 pandemic: lessons from a polarized scenario in Brazil

**DOI:** 10.3389/fpsyg.2024.1310594

**Published:** 2024-04-10

**Authors:** Karla Gonçalves Camacho, Daniella Campelo Batalha Cox Moore, Maria de Fátima Junqueira-Marinho, Saint Clair Gomes Junior, Adriana Teixeira Reis, Dimitri Marques Abramov

**Affiliations:** ^1^National Institute for Women, Children, and Adolescent Health Fernandes Figueira, Department of Pediatrics, Oswaldo Cruz Foundation (Fiocruz), Rio de Janeiro, Brazil; ^2^State University of Rio de Janeiro, Department of Perinatology, Rio de Janeiro, Brazil; ^3^Fluminense Federal University, School of Medicine, Internal Medicine Department, Niterói, Rio de Janeiro, Brazil; ^4^National Institute for Women, Children, and Adolescent Health Fernandes Figueira, Clinical Research Department, Oswaldo Cruz Foundation, Rio de Janeiro, Brazil; ^5^National Institute for Women, Children, and Adolescent Health Fernandes Figueira, Teaching Department, Oswaldo Cruz Foundation, Rio de Janeiro, Brazil; ^6^National Institute for Women, Children and Adolescent Health Fernandes Figueira Laboratory of Neurobiology and Clinical Neurophysiology, Oswaldo Cruz Foundation, Rio de Janeiro, Brazil

**Keywords:** resistance to lockdown, COVID-19, denialism, economic insecurity, pandemics

## Abstract

**Introduction:**

The COVID-19 pandemic led many countries to adopt strict measures aimed at reducing circulation of the virus and mitigating the burden on health services. Among these, the lockdown (social distancing/confinement) was probably the most controversial and most widely debated, since it affected the population’s daily life abruptly, with consequences for people’s emotional state and the operational logic of various economic sectors.

**Objective:**

Analyze the relationship been Brazilians’ opinions on lockdown during the pandemic and individual, sociodemographic, and belief characteristics.

**Methods:**

We conducted an online survey to evaluate Brazilians’ opinions on the lockdown during the COVID-19 pandemic. We prepared a questionnaire with questions on sociodemographic aspects and individuals’ points of view toward the lockdown. We sent a link for the survey through social media and encouraged participants to also share the link in their respective social networks, as a snowball sample. Cluster analysis was performed to identify different opinion profiles. Cluster Analysis is a multivariate approach that aims to segment a set of data into distinct groups, using some classification criteria.

**Results:**

From April to May 2021, the link received 33,796 free participations via social networks from all over Brazil. We analyzed data from 33,363 participants. Pro-lockdown opinions predominated in most of the sociodemographic strata. Cluster analysis identified two groups: pro-lockdown, aligned with the scientific recommendations, and anti-lockdown, characterized by economic insecurity and denialism. Anti-lockdown participants downplayed the pandemic’s seriousness and believed in unproven measures to fight SARS-CoV-2. However, these same participants were afraid of losing their jobs and of being unable to pay their bills. In general, participants did not believe in the feasibility of a lockdown in Brazil or in the efficacy of the prevailing government administration’s measures.

**Conclusion:**

The study identified a lack of consensus among participants concerning lockdown as a practice. Issues such as disbelief in the pandemic’s seriousness, denialism, and economic insecurity were important in the determination of the profiles identified in the study. Denialism is believed to have been a subjective defense against the economic problems resulting from social control measures and the lack of adequate social policies to deal with the pandemic. It was also highlighted that political polarization and the lack of central coordination during social distancing are crucial aspects. The variation in results in different locations highlights the diversity of the Brazilian scenario. By analyzing Brazilians’ opinions about the lockdown, considering individual characteristics, the study seeks insights to face the pandemic and prepare for future crises, contributing to more effective public health strategies.

## Highlights

- The resistance was marked by denial of the pandemic’s seriousness.

## 1 Introduction

The COVID-19 pandemic raised a series of unprecedented global challenges and resulted in many tragic losses in various areas, generating multiple stress factors, an impact on young people’s mental health, and serious interruptions in health services (Pan American Health Organization and World Health Organization, 2022). The pandemic led various countries to adopt strict health measures aimed at reducing circulation of the virus and mitigating the burden on health services. The measures taken to attenuate the pandemic’s impact included lockdown, vaccine development, economic support, and efforts to promote mental health and community resilience. Among these, lockdown (social distancing/confinement) was probably the most controversial and hotly debated, since it affected the population’s daily reality abruptly with consequences for people’s emotional state and the operational logic of various economic sectors (Noronha et al., 2020). The restrictions, initially planned to last for a brief period, were maintained for nearly 2 years due to the increase in infection rates and new SARS-CoV-2 variants (Pawlak and Sahraie, 2023). Although lockdown is a radical measure, it is considered one of the most effective for controlling the spread of contagious diseases, especially during pandemics. The practice consists of suspending transportation, commerce, and services, only maintaining the functioning of essential services such as healthcare, food, and public security, among others (Hamzelou, 2020).

Public health authorities such as the World Health Organization and the United States Centers for Disease Control (CDC) concentrated efforts in the early phase of the pandemic on combatting the spread of the novel coronavirus by encouraging social distancing as an effective nonpharmacological measure against spread of the disease (Kharroubi and Saleh, 2020; Mégarbane et al., 2021). However, some authors contend that lockdown’s efficacy is concentrated in early implementation and gradual phaseout of social distancing, more than in its rigorous execution (Mégarbane et al., 2021). Some Brazilians believed that the broad restrictions implemented by mayors and governors in the fight against COVID-19 not only would fail to slow the rise in cases, but they would also have significant harmful effects, such as worsening poverty, family crises, increased street crime, mental health problems such as depression, and increases in harmful behaviors such as suicide and substance abuse (Barbosa et al., 2020).

Brazil, as other countries, adopted social distancing measures such as restriction on the entry of foreigners into the country via airports, bans on in-person classroom activities in schools, and cancelation of appointments with healthcare professionals and even of elective surgeries. However, unlike in other geographies, in Brazil, social distancing and all the measures to mitigate the pandemic were marked by politicization, fueling polarization in the opinions for and against the measure, and lack of central coordination, with states, municipalities, and civil society left to seek solutions to the strangulation of local health services and the lack of reliable data on prevalence, incidence, or mortality. This troubled context meant that Brazil experienced different scenarios in the pandemic’s control. Cities such as Araraquara in the interior of São Paulo state showed low incidence, while others such as Manaus, capital of Amazonas state, saw its health system totally suffocated and with shortage of basic inputs such as oxygen, caused by COVID-19 (Bocchini, 2021; Malta et al., 2021). Governments across the world have faced complex challenges, where individual responses to prevent transmission have been as crucial as government actions. Effective communication strategies to inform the public about preventative measures were essential, as was additional support to address the economic crisis (Anderson et al., 2020).The arguments in favor of containment measures followed recommendations by international bodies such as the International Monetary Fund (IMF), which recommended lockdown during the COVID-19 pandemic, contending that economic recovery could occur more quickly after the lockdown (Caselli et al., 2020). Meanwhile, the arguments against social distancing measures followed the classic playbook of neoliberal thinking, according to which the State should not interfere in people’s individual routines and that the economies of emerging countries such as Brazil would be unable to guarantee financial aid for long periods, for the citizens affected by the economic downturn. Some 3 years after the start of the pandemic, the World Health Organization declared the end of the Public Health Emergency of International Concern (PHEICI) referring to COVID-19 (Pan American Health Organization and World Health Organization, 2023), on May 5, 2023. Currently, there are still disparities in the impact of COVID-19 on the financial markets of developed and developing countries. In the former, the negative influence on supply, demand and economic stability stands out, while in the latter, impacts on confidence, expectations and consumption patterns are highlighted (Zhao et al., 2023).

The political polarization and the way that the federal government dealt with the health measures against COVID-19 left deep marks on Brazilian society. Future research will need to study the consequences of these restrictive measures to understand the rationale and efficacy of social distancing policies on the economy and especially on global mental and public health. We find that even so long into the pandemic, there is still heavy debate on the application of lockdown in society, including in Brazil, where there is no consensus on the issue.

This study addresses the complexity of the COVID-19 pandemic in Brazil, highlighting losses, stress factors and imposed measures. Political polarization and the lack of central coordination during social distancing are crucial aspects. The variation in results in different locations highlights the diversity of the Brazilian scenario. By analyzing Brazilians’ opinions about the lockdown, considering individual characteristics, the study seeks insights to face the pandemic and prepare for future crises, contributing to more effective strategies in public health. Our objective was to analyze the relationship between Brazilians’ opinions on lockdown during the pandemic and individual, sociodemographic, and belief characteristics, using an online exploratory survey, as well as to discuss the possible correlations with the population’s beliefs on lockdown, which can help to confront future pandemics.

## 2 Materials and methods

### 2.1 Design and subjects

This was a cross-sectional exploratory study of the relationship between sociodemographic aspects, beliefs on the pandemic, and opinions on lockdown, using an online questionnaire answered after agreeing to the online free and informed consent form. The answers were completely anonymous. The study was approved by the Institutional Review Board of the IFF (CAAE: 45053221.1.0000.5269) through review no. 4.640.611 on April 9, 2021, in keeping with Brazilian and international legislation on research involving human subjects.

In April 2021, the researchers sent the link for completing the questionnaires to their respective contact lists (family, friends, professional contacts) and followers in social media and networks. After participants completed the questionnaire, they were then encouraged to resend the link to their own contacts and followers on social media and networks, resembling a snowball process. This strategy aimed to increase the survey’s dissemination and expand its target public. Research participants were considered fit to complete the questionnaire if they confirmed that they were born in Brazil and were 18 years or older. Research participants were excluded by the researcher if they did not take a position either for or against the lockdown. During the data analysis, measures were implemented using software that identified possible duplicate records or inconsistencies in fields that were considered essential, such as age or place of birth.

### 2.2 Questionnaire

A questionnaire was produced in Google Forms with 38 questions (see complementary information 01). The last three questions were open-ended, allowing participants to freely express their opinions on lockdown. The other 35 questions were closed, of the yes-or-no type, or in the Likert scale format with the following possible answers: totally agree, agree, neither agree nor disagree, disagree, totally disagree.

In the questionnaire’s first section, we discuss the principal sociodemographic markers, namely: sex, family income, schooling, state of residence, work sector (public, private, etc.), professional field, COVID-19 history, and COVID-19 vaccination history.

Another section addressed opinions on the pandemic and lockdown, introduced by a text that defines lockdown as an “emergency measure to avoid the pandemic’s spread, suspending various activities (cultural, entertainment), closing of businesses and nonessential services (shopping centers, stores in general, bank services, etc.), maintaining nonessential workers at home (industries, administrative, etc.), and blocking free transit of persons in risk areas.” The first question in this section was categorical and was answered objectively as “against” or “for” lockdown.

The selection of variables in this study was carefully strategized to align with the research objectives. Variables were chosen based on their relevance to the study theme, focusing on psychological, economic, and social dimensions related to participants’ attitudes toward pandemic-related confinement measures. The decision was guided by a thorough literature review to ensure theoretical grounding and the representativeness of the analysis.

### 2.3 Data and statistical analysis

The sample was described via absolute and relative frequencies of the target variables. The Cluster analysis, based on the two-step method, was used to classify participants with similar characteristics and response patterns. Cluster Analysis is a multivariate approach that aims to segment a set of data into distinct groups, using some classification criteria. The goal is to create data partitions where homogeneity is maximized within each group, while heterogeneity is maximized between groups (Sneath and Sokal, 1973). The technique allows working simultaneously with large datasets and categorical and numerical variables. The algorithm provides for conducting a nonhierarchical step, resembling k-means, and a grouping hierarchy to form homogeneous Clusters.

Participants were classified according to maximum likelihood, and the number of Clusters was defined by the Bayesian information criterion (BIC) and silhouette coefficient. Researchers commonly use the Bayesian Information Criterion (BIC) to examine and compare the adequacy of models in several areas of statistical simulation, including multiple regression and generalized linear models (Raftery, 1998).The silhouette coefficient assesses the resulting Clusters’ cohesion and discrimination, varying from −1 to +1, where positive values greater than 0.5 indicate reasonable partition of the sample between the Clusters, and values less than 0.2 indicate that the data do not have a cluster structure. The final number of Clusters was defined in this study based on the maximum observed value of the silhouette coefficient between the different partitions of the dataset.

We summarized each group’s profile considering each question (sociodemographic, opinions, and views) as a specific characteristic of each Cluster. We attributed each characteristic when the difference of frequencies for a given question was greater than 50% (number of Clusters) between the Clusters, while simultaneously convening more than 50% of the cases in the same Cluster. In questions of the Likert scale type, we only considered the frequencies of agreement for such characterization.

## 3 Results

The study included 33,363 participants from April 8 to 17, 2021, after applying the eligibility criteria.

The variables considered for the cluster analysis made it possible to identify two groups, as described in Table 1. Cluster 1 is characterized by having the majority of people in favor of lockdown, being female, having completed higher education and with a professional profile in the area of education., arts, culture and social communication. On the other hand, Cluster 2 is characterized by having the majority of people opposed to the lockdown, male, working in the private sector and with a professional profile related to manual work, commerce, industry, security and armed forces. Based on this description, cluster 1 will be called in favor of lockdown and Cluster 2 as being against it.

**Table 1:** Sociodemographic variables, professional fields, and opinions on lockdown in the two Clusters identified in the study population.

	Cluster 1 (*N* = 23.632)	Cluster 2 (*N* = 8.258)
**Opinion on lockdown** For Against	23,336 (98.7%) 296 (1.3%)	291 (3.5%) 7,967 (96.5%)
**Gender** Female Male Nonbinary/not informed	16,494 (69.8%) 7,007 (29.7%) 131 (0.6%)	3,578 (43.3%) 4,629 (56.1%) 51 (0.6%)
**Age (years)** 18 to 39 40 to 59 60 or older	6,669 (28.3%) 10,958 (46.6%) 5,906 (25.1%)	2,236 (27.2%) 3,982 (48.5%) 1,988 (24.2%)
**Monthly income (BRL)** ≤ 3,499 3,500-8,499 ≥ 8,500	4,616 (19.7%) 8,599 (36.7%) 10,202 (43.6%)	1,977 (24.2%) 2,564 (31.4%) 3,636 (44.5%)
**Schooling** Primary or less Complete secondary Complete university	193 (0.8%) 3,115 (13.2%) 20,229 (85.9%)	212 (2.6%) 1,771 (21.5%) 6,237 (75.9%)
Work sector* Private sector Public sector Other	8,445 (36.4%) 9,277 (39.9%) 6,702 (28.8%)	4,434 (54.6%) 2,059 (25.4%) 2,100 (25.9%)
Professional field* Unemployed Homemaker Health Manual labor Education Arts, culture, and similar Social communication Commerce Services Administrative Industry Science and technology Security Armed forces Other	1,642 (7%) 707 (3%) 4,279 (18.2%) 196 (0.8%) 7,672 (32.5%) 1,846 (7.8%) 981 (4.2%) 710 (3%) 1,864 (7.9%) 1,470 (6.2%) 637 (2.7%) 1,918 (8.1%) 143 (0.6%) 68 (0.3%) 2,296 (9.7%)	629 (7.6%) 297 (3.6%) 1,509 (18.3%) 133 (1.6%) 890 (10.8%) 225 (2.7%) 115 (1.4%) 1,021 (12.4%) 874 (10.6%) 640 (7.8%) 484 (5.9%) 717 (8.7%) 219 (2.7%) 278 (3.4%) 888 (10.8%)
**COVID-19 infection** Yes No	10,101 (42.8%) 13,506 (57.2%)	4,874 (59.1%) 3,371 (40.9%)
**Vaccinated against COVID-19** Yes No	5,809 (24.7%) 17,746 (75.3%)	1,652 (20.1%) 6,576 (79.9%)

& - approximate values.

* Multiple choices were allowed for the work sector and professional field, so the total may exceed 100%.

Table 1 – Sociodemographic variables, professional fields, and opinions on lockdown in the two Clusters identified in the study population.

**Figure 1:**
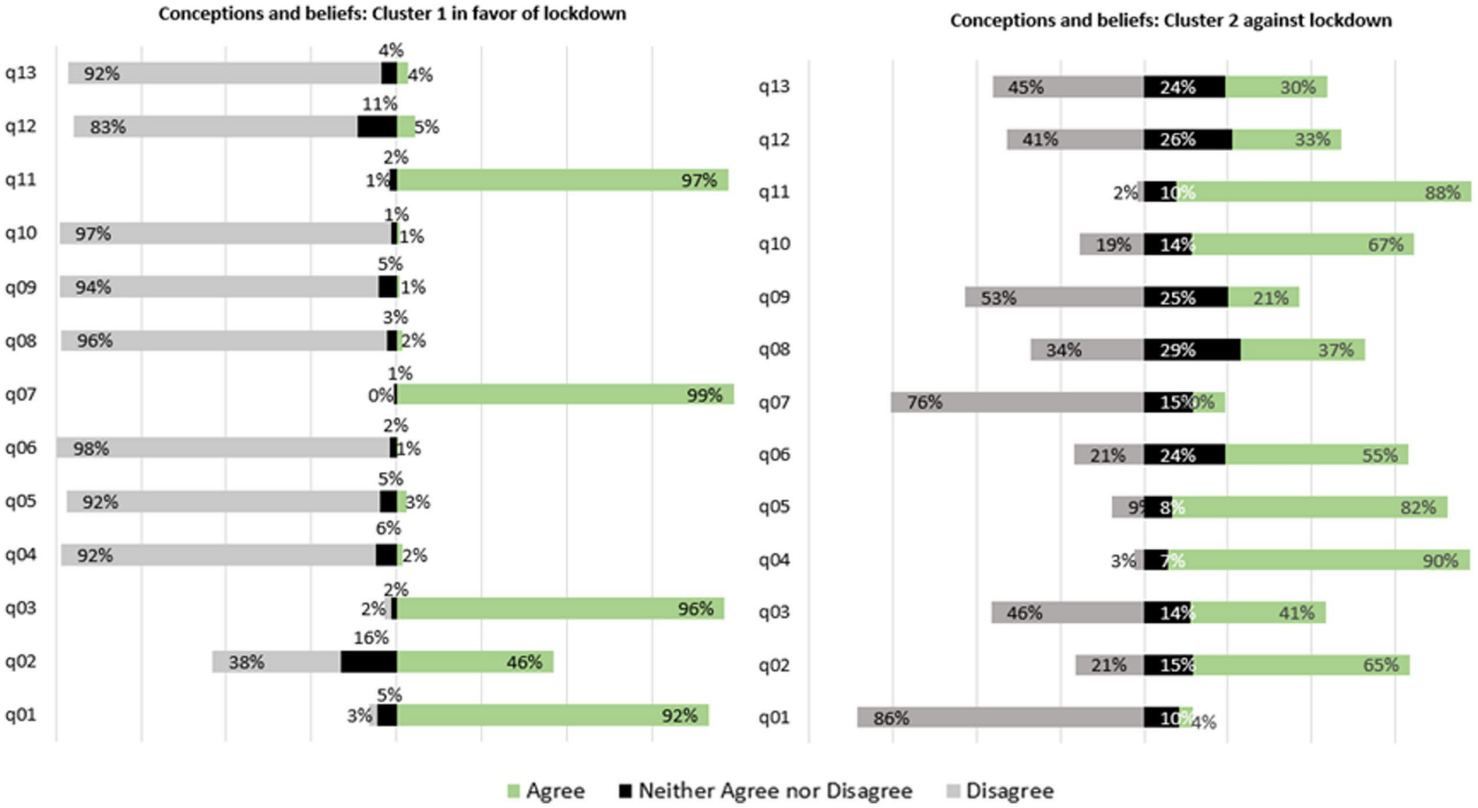
Conceptions and beliefs from Cluster 1 and Cluster 2. Questions:q01 - The financial harm from lockdown can be resolved later. It’s necessary to save lives; q02- Lockdown is not possible because Brazil lacks an adequate emergency financial relief; q03 - The economy will not improve unless the pandemic is controlled; q04 - The harm to the economy from a lockdown would be irreparable; q05 - People should not be prevented from coming and going under any circumstances during the pandemic; q06 - We could solve everything with prophylactic treatment (ivermectin, hydroxychloroquine…); q07 - I believe that lockdown will help relieve the overload on health services; q08 - Herd immunity would be the best solution. Everyone that catches COVID would become immune, and the disease would stop spreading; q09 - I think everyone’s fate is sealed: whoever dies from COVID is bound to die anyway; q10 - I do not believe the pandemic is as serious as the press claims; q11 - I believe that I have taken effective measures to contain the pandemic; q12 - I believe other people have taken effective measures to contain the pandemic; q13 - I believe that the government has taken effective measures to contain the pandemic.

To address study participants’ conceptions and beliefs that can potentially affect their opinions on lockdown, questions were asked, and the results were analyzed according to the Likert scale (Figure 1). Numeric data of the data from this study are avaliable in Supplementary Table S1 - Beliefs and thoughts on the pandemic and lockdown in the two Clusters identified in the study population and Supplementary Table S2 - Fears, feelings, and behaviors toward the pandemic and lockdown in the two Clusters identified in the study population as supplementary materials.

The first four questions in Figure 1 were related to the influence of Lockdown on the economy. The belief that the financial harm from lockdown could be resolved later was the one that showed the greatest disagreement between the two groups (q01 in Figure 1). The belief that the harm to the economy caused by the lockdown would be irreparable (q02 in Figure 1) did not show consensus between the two groups. The group against lockdown (Cluster 2) believes that the damage is irreparable, while the group in favor of lockdown believes that the damage to the economy can be remedied later. Some disagreement also occurred regarding the belief that pandemic should be controlled first for the economy to improve, while 96.1% of those from cluster 1 disagree with this belief, almost half of those in cluster 2 agreed. Those from Cluster 2 seem to understand that it was not possible to wait to address the impact on the economy and that this harm has also the potential to claim lives. Both groups believe that Brazil’s lack of an adequate emergency support policy to make the lockdown feasible.

The questions 5 (q05) and 6 (q06) adressed the sense of autonomy in the group in favor of lockdown and in the group against lockdown (Figure 1). Lockdown interferes with a moral value that is important to many people, which is the right to come and go. Those in Cluster 2, for the most part, agreed that no one should have this freedom restricted (q05 Figure 1), that is, the individual’s autonomy should not be harmed. In April 2021, when this online survey was conducted, vaccination to prevent COVID-19 had already started in Brazil, but it was not for everyone. The access followed a priority schedule that started with the vaccination of the elderly and healthcare professionals. The use of medicines without scientific proof such as irvemectin and hydroxychloroquine for the prevention and/or treatment of COVID-19 (q06 in Figure 1), was rejected by the majority of those in cluster 1 but accepted in 55% of those in cluster 2 and may have been a reflection of an attempt by the individual to express their autonomy, assuming responsibility in the face of the slow and faltering supply of immunobiologicals with high efficacy against COVID-19. For Cluster 2, whose components were mostly against the lockdown, the use of these medications may have give them cognitive support to face the fear of being infected and mantain free circulation.

Questions 7, 8, 9, and 10 (Figure 1) addressed whether the severity of the pandemic was underestimated. In addition to believing in medicines without proven efficacy, another pillar that helped those against the lockdown to deal with the fear of becoming infected while maintaining circulation was the denial of the seriousness of the pandemic. Cluster 2 underestimates the severity of the pandemic (q10 in Figure 1) and does not believe that the lockdown would relieve the burden on health services (q07 in Figure 1). In line with the desire or need to continue circulating to move the economy and maintain one’s jobs, believing that this behavior can help form herd immunity through natural imunity (q08 in Figure 1) seems congruent. Unfortunately, unlike herd immunity obtained through immunization, that obtained through natural immunity has a serious side effect that is the death of many. The death as a destiny expressed in the belief that whoever died was bound to die anyway was rejected by the majority of the two groups, however 21% of those in Cluster 2 agreed (q09 in Figure 1). Therefore, for some of those in cluster 2, deaths related to SARS-CoV-2 infection were underestimated, relegating them to the tragic fate of each individual, since one of the recommended measures to avoid these deaths was lockdown, a measure rejected by this group.

The fight against the pandemic required precise attitudes and behaviors by individuals, society in general, and government to control the spread of the virus and gradually decrease the number of COVID-19 cases and deaths. Participants were asked what they thought about their own attitudes (q11 in Figure 1), the attitudes of others (q12 in Figure 1), and the government’s position (q13 in Figure 1) to help contain the pandemic. Most participants in Cluster 1 believed that they were doing their part, but they did not think the government or other people were doing so. This view was also seen in Cluster 2, but more heterogeneously.

**Figure 2:**
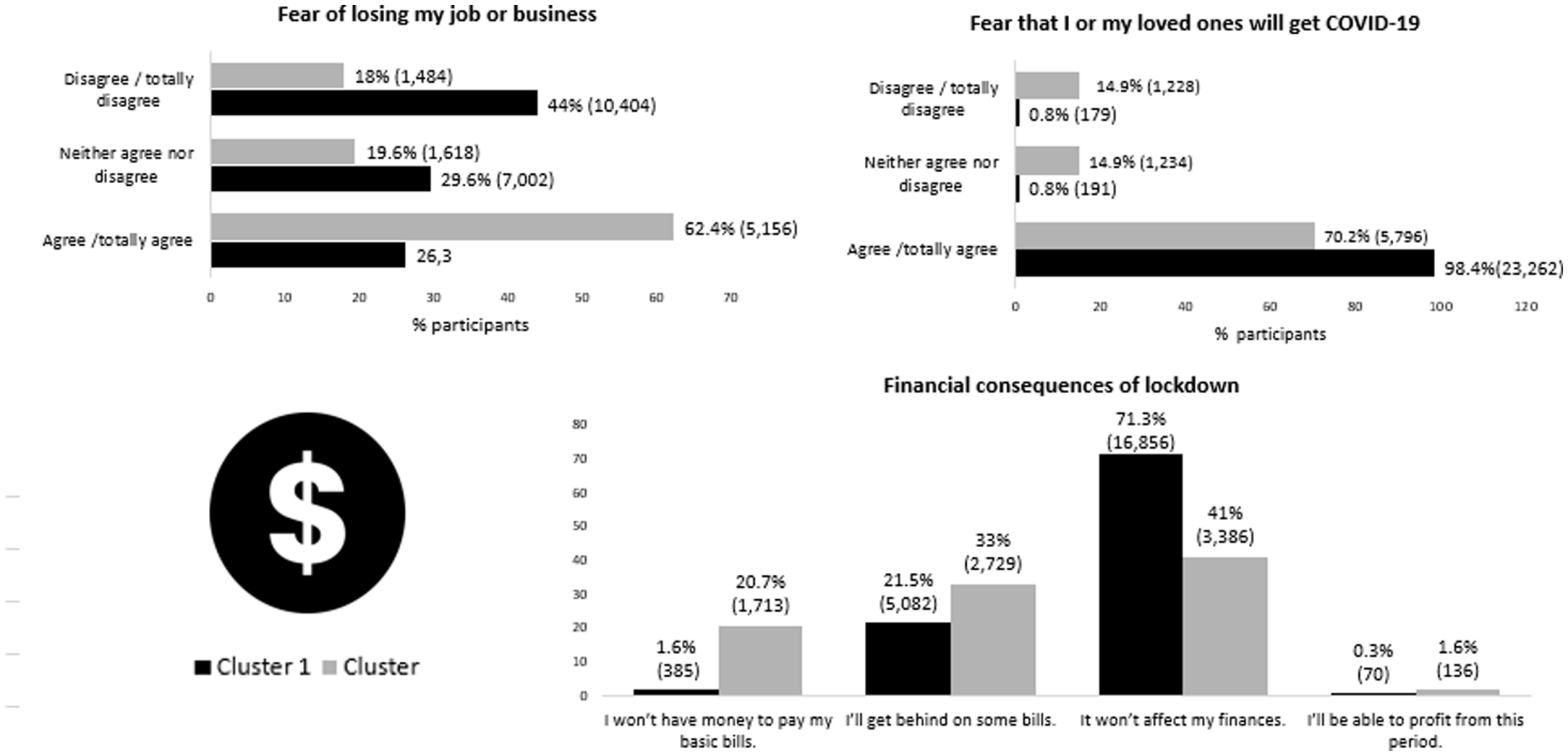
Fear of impact in the health and jobs during pandemics.

**Figure 3:**
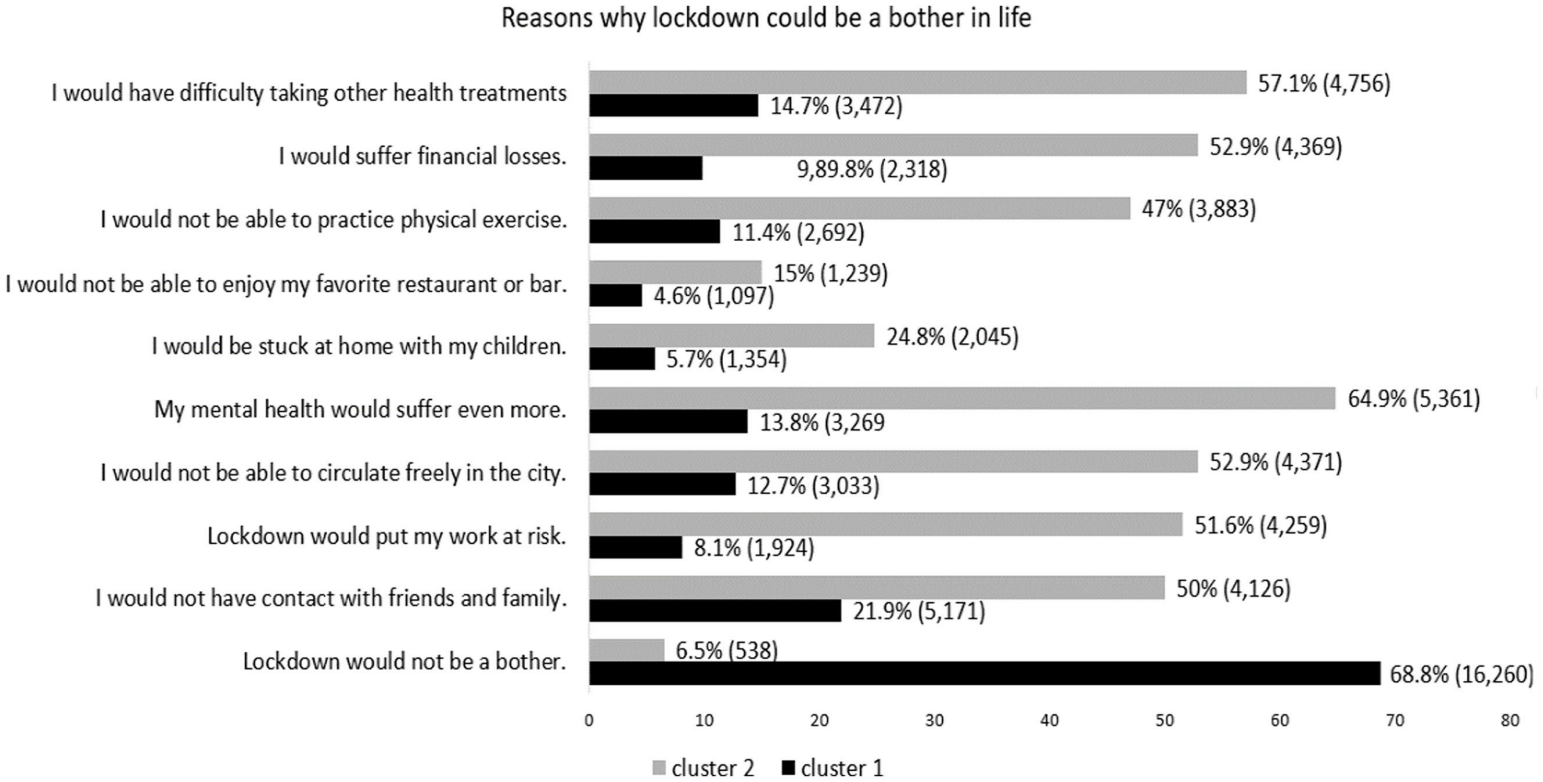
Reasons why lockdown could be a bother in life.

The fears and feelings in relation to pandemic and lockdown were also assessed. Despite the denial of the pandemic observed, the two groups showed consensus when expressing the fear of becoming infected or that one of their loved ones had COVID-19 (Figure 2). However, although the fear of impact on work was expressed by the majority of those who make up cluster 2, only 26% of those belonging to Cluster 1 expressed this fear. It is possible that participants in Cluster 1 are linked to jobs with greater job security and stability and Cluster 2 is more linked to jobs with greater precarious social security or linked to individuals who are their own bosses such as independent professionals and entrepreneurs. This hypothesis, however, cannot be confirmed in this study as the nature of the employment relationship was not studied. The current information is that the majority of those in Cluster 2 were in the private sector, while in Cluster 1 the distribution between the private and public sector was more homogeneous (Table 1). In support of this impression of financial instability related to Cluster 2, Figure 3 shows that 53% of participants in Cluster 2 believed that they would not have money for basic bills or be able to pay some bills, a concern that was less present among those in Cluster 1.

Figure 3 shows how individuals belonging to the two Clusters understand that the lockdown could disrupt their lives. An important concern observed among those who make up cluster 2 is related to the impact on mental health in the face of a lockdown, while only 13.8% of those who belong to Cluster 1 demonstrated this concern. Other aspects observed regarding the fear of lockdown interfering in the participants’ lives were more prominent in Cluster 2 than in 1, including: difficulty with other health treatments, financial losses, not moving freely, putting the work at risk, not spending time with friends and family and not being able to exercise. There are reports in previous studies that indicate a significant decrease in physical activity levels during COVID-19 (Jagim et al., 2020; Taheri et al., 2023). It is not possible in this study to determine whether those in Cluster 1 did not resent the restriction on physical activity or whether they found other forms of indoor exercises.

**Figure 4:**
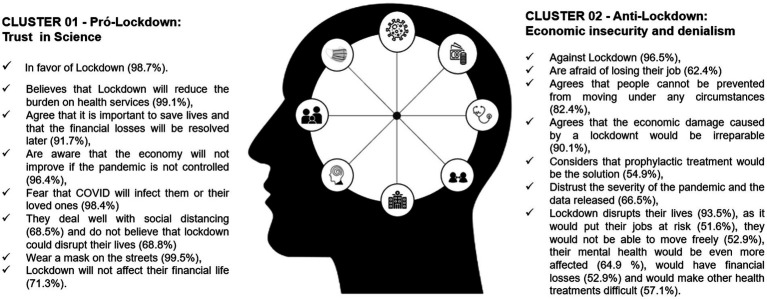
Profiles of the two Clusters. The principal characteristics in each of the Clusters were listed to understand their profiles.

Recommendations for dealing with the COVID-19 pandemic meant that individuals had to deal with social distancing and wearing a mask in public. When asked to confirm if they dealt well with social distancing, that is, the need to maintain a distance of around 1.5 m from other people, among 68.5% (16,182) of those in Cluster 1 and 31.4% (2,592) of those in Cluster 2 agreed; 16.8% (3,966) of cluster 1 and 16.7% (1,377) of cluster 2 were indiferente; And, 14.7% (3,484) of those in Cluster 1 and 51.9% (4,289) of those in Cluster 2 responded that they disagreed. Regarding the use of masks, around 99.5% (23,504) of those in cluster 1 and 75% (6,255) of those in cluster 2 agreed that they always wear masks in public. 0.2% (57) of those in Cluster 1 and 7.4% (610) of those in Cluster 2 were indifferent; And 0.3% (71) of those from Cluster 1 and 16.9% (1,393) disagreed. Therefore, we observed that those in Cluster 2 had more difficulty dealing with social distancing and were a little less adhering to the use of masks in public than Cluster 1. The overwall sadness if lockdown happens were agreed by only 16.5% of those in Cluster 1, but by the majority (89.8%) of those in Cluster 2.

This study shows that those who are against or in favor of lockdown in the face of health emergencies have different beliefs and mindsets that can be strongly driven by issues related to financial insecurity.

## 4 Discussion

The study confirmed the hypothesis of polarization in the Brazilian population on lockdown during the COVID-19 pandemic. The discussions and debates have continued concerning the positive and negative impacts of this measure for the Brazilian reality even after the World Health Organization declared the end of the health emergency (Pan American Health Organization and World Health Organization, 2023). Cluster 1 represented the great majority of the study population in all sociodemographic strata. It consisted of participants who supported lockdown and believed that it would reduce the number of deaths, avoid a breakdown in the health system, and foster economic recovery. Cluster 1 consisted of people that were less vulnerable in the social context since they enjoyed greater economic protection and more schooling and could thus follow the recommendations for prevention and social distancing. This Cluster was thus identified as the “pro-lockdown segment according to the scientific evidence” (Table 1).

Meanwhile, Cluster 2 consisted mostly of people that were anti-lockdown, and this Cluster represented approximately one third of the study population. This group did not believe in the seriousness of the pandemic as announced by the press, thus revealing a denialist behavior, concern over individual freedoms, and a propensity to believe in herd immunity and baseless pharmacological measures to prevent COVID-19. Concerns over jobs and the economy predominated in this Cluster. This was the “anti-lockdown group: denialism and economic insecurity” (Table 1).

Denialism is a self-defense mechanism that consists of denying exterior reality and replacing it with a fictitious one. The mechanism has the capacity to deny parts of unpleasant and undesirable reality through the fantasy of satisfying desires or behaviors. “In the Freudian sense (…) it tends to be reserved to designate the refusal to perceive a fact that imposes itself on the outer world” (Laplanche et al., 2001).

Participants in Cluster 2 tended to downplay the pandemic’s risk, together with misinformation and discrediting of health experts, an issue that has been the object of discussion in Brazil and elsewhere in the world (Vasconcellos-Silva and Castiel, 2020; Paul et al., 2021). Skepticism toward the pandemic’s seriousness among people that are against lockdown may be a disadvantage for awareness-raising of society, probably fed by social media and fake news (Paul et al., 2021). Mistrust of evidence-based recommendations became a dangerous behavior, since denying the truth is not merely a personal position, but a public health issue and a challenge for society in general.

Denialism by many people in the COVID-19 pandemic may be a reflection of weak governance which points to the existence of limited alignment between the scientific evidence and prevention policies by the Brazilian government administration. Experts have contended that when the relationship between health and public policies is misaligned, scientific evidence alone will be insufficient to develop action plans. The issue is to know how to address and overcome this dichotomy (Horton, 2020). Weak governance is a risk to public health, especially in more serious moments such as during pandemics. A study argued that the Brazilian federal government’s pandemic response was weak in five risk governance parameters that are essential in a response to health crises: risk communication, data transparency and accessibility, negotiation between actors, social cohesion and participation public and decisions based on technical criteria and scientific evidence, resources and contexts (Di Giulio et al., 2023). Adherence to social distancing measures during a pandemic may be influenced by a combination of factors that affect people’s perceptions and attitudes toward such measures. Some factors that may favor adherence to social distancing measures are clear and transparent communication, trust in scientific proof, strong leadership, financial and social support, support from authority figures and influencers, examples of success, community involvement, and access to basic resources (Bavel et al., 2020).

A scoping review showed that participants were more likely to accept lockdown when there was a perception of risk from the disease and when communications were articulated by authorities in whom they trusted (Ranieri et al., 2023). In Brazil, the federal government showed a notoriously denialist stance toward the pandemic that discouraged mask wearing and social distancing (Idrovo et al., 2021). This suggests less disagreement on the government’s effectiveness in managing the pandemic in the anti-lockdown Cluster, which may mean greater political alignment between this group and the government administration in office at the time. Another factor observed in the anti-lockdown Cluster was greater sadness over the possibility of lockdown and a feeling of deprivation of freedom. The relationship between perceived coercion and lockdown has been assessed in some studies, and a review has suggested that people who feel less control over their lived experiences suffered greater anxiety, depression, and a feeling of imprisonment (Ranieri et al., 2023). Another factor potentially contributing to greater sadness in the anti-lockdown group may suggest suffering linked to neoliberal ideologies that can contribute to the anguish many people experienced during the pandemic. The neoliberal ideology encourages a kind of individualism that diminishes the feeling of collectiveness, community, and social connectedness, which are important for engagement in public policies such as lockdown (Zeira, 2022). Zeira et al. highlighted that this ideology contends that individualism is a desired moral characteristic, and that asking for help, especially financial help, would be contrary to the principles in which they believe. Thus, becoming dependent on government aid is inconsistent with these principles. With total faith in the free market, these participants may not been able to find a way out of their financial chaos in case of a lockdown, which may have generated internal conflicts that increased their suffering. A study found that higher neoliberal anti-government interference beliefs were associated with lesser life satisfaction during pandemics when the limits of government power was brought to the forefront of public discourse and daily experience (Card and Hepburn, 2023). The economy, marked by neoliberal ideology, presented difficulties in balancing itself even in the face of a temporary suspension of circulation, demanding circulation even when such circulation demonstrably promoted illness (Nunes, 2020). The advance of the pandemic found in Brazil a political environment aligned with neoliberal ideology with speeches that mixed defense of the free market and at the same time disseminated misinformati to promote the free circulation (Machado, 2021; Paula et al., 2023).

Another interesting aspect was the predominance of women in the pro-lockdown Cluster and of men in the other Cluster, which raises the issue of how the view on lockdown can be affected by gender. Studies on gender and health in Brazil suggest that women are more concerned than men about healthcare, and that women attend health units more and undergo more treatments (Pinheiro et al., 2002). It has been discussed that care is historically identified with female behavior in Brazil, while the construction of the male gender starts from a place of strength, leaving no room for healthcare (Griffith et al., 2020; Medrado et al., 2021). The gender imbalance in the two Clusters may have resulted from the discourse of previous Brazilian government authorities who reinforced beliefs in male supremacy in society and in the patriarchy, perpetuating power relations and reinforcing conservative values and myths, but with a strong appeal to the “national” identity (Cunha, 2014; Duarte and da Silva et al., 2021; Figure 4).

Although there were fewer people than in the pro-lockdown Cluster, 70% of participants in the anti-lockdown Cluster mentioned their fear of catching COVID-19. The expectation is that people will adopt protective behaviors in response to the perception of risk. However, a study has revealed that there is no correlation between the perception of risk and the practice of handwashing (Trifiletti et al., 2022). Mistaken beliefs have the same potential to lead to risky behaviors. On the one hand, unlike the pro-lockdown Cluster, we found that most of the anti-lockdown Cluster consisted of people who had already had COVID-19, which may explain the fact that they downplayed it. However, the paradox is perceptible: the fear of being infected is more prevalent than the number of people who had already caught COVID-19, which shows the fear of reinfection. The expression of this conflict suggests that denialism may be a manifestation of psychological defense, which is denial itself; it emerges when the self is unable to cope with reality that assaults it and it needs to construct fantasies to protect itself from this reality (Beilin, 1982). Denial is based on five discursive strategies that, used together or separately, result in pseudoscientific discourse. Malinverni and collaborators believe that three of these strategies highlight how the arguments of American experts contribute to supporting the denialism of Bolsonaro and his followers: selective choice of scientific data out of context to suggest error; use of experts whose opinions are not aligned with the established scientific consensus; and reference to isolated articles that challenge prevailing opinion as a way of discrediting the entire field (Malinverni and Brigagão, 2020). Denial occurs, for example, when a fatal disease is diagnosed (Beilin, 1982). However, denialism may be merely the surface that covers the true motivation, namely economic insecurity. Called financial insecurity in an environment of weak governance. Thus, with financial fears unaddressed, they deny the pandemic approach that only looks at health issues.

Economic insecurity appeared to be a key element in the opposition to lockdown. The characteristics of the anti-lockdown profile were fear of losing one’s job or business; the individuals were employees of private companies and thus more vulnerable to being laid off in Brazil. The lack of conditions to conduct the lockdown (because the government lacked an adequate emergency relief policy) was a consensus even in the pro-lockdown Cluster, who did not appear vulnerable to the financial issue and were favorable to the lockdown.

Although the restrictive measures may decrease transmission of the virus, their impact on the economy is undeniable. Reports by the World Bank showed that the pandemic pushed millions of people into extreme poverty and may still leave scars that will force activities and earnings below their pre-pandemic levels (The World Bank, 2021). Studies showed an increase in food insecurity after the pandemic (Pereira and Oliveira, 2020; Manfrinato et al., 2021). There were signs that the impacts were particularly heavy in countries with weak economies, mainly in the informal sector, due especially to the limited emergency relief (Chackalackal et al., 2021). To restore pre-pandemic normality, it is crucial to implement effective, safe, accessible, and widely available measures, including vaccination, addressing persistent challenges posed by COVID-19 (Dergaa et al., 2021). For social distancing measures to be successful, it was important for them to be complemented by social protection measures that guaranteed minimum income, food, and access to basic services (Economic Commission for Latin America and the Caribbean and Pan American Health Organization, 2020). However, if SARS-CoV-2 transmission were not controlled, the economies would not recover (Pan American Health Organization and World Health Organization, 2020). Still, the beliefs of people in the anti-lockdown Cluster revealed a movement against the latter recommendation.

Implications of the study suggest the need for holistic and tailored approaches to address the population’s diversity of opinions and concerns during public health crises. Practical actions include specific communication strategies, diversity-sensitive public policies and effective integration between science and government policies.

The principal limitation identified in this study was the convenience sample, which may not have reflected the opinions of Brazilians in general, since it was not proportionally representative of the national sociodemographic profile. However, all the socioeconomic strata were represented, so we had the qualitative potential to discuss beliefs, views, and behaviors related to opinions on lockdown. For example, it is worthwhile to underscore the unequal participation of women and individuals with high income and more schooling, who formed the majority of this study’s sample. This problem has been seen frequently in online surveys, as discussed in previous studies (Spyer, 2017). A limitation of this study was not having characterized the employment relationship in which those responsible for the participant’s family income were subject. This delimitation could have enabled reflections on economic fragility in the face of health emergencies or other crises that affect the economy. Another limitation was not having addressed the political alignment with President Jair Bolsonaro, who repeatedly demonstrated that he was against the lockdown.

## 5 Conclusion

The study identified polarization on agreement with lockdown among the participants, suggesting the urgency of building a middle road, which will only be possible if we understand that it is not about a fight between good and evil, but rather about people who share the same desire to survive. With this understanding, to perceive issues related to the anti-lockdown position such as denialism of the pandemic’s seriousness, the feeling of being deprived of freedom, economic insecurity, and the respective psychological underpinnings may help contribute to more effective communication strategies. The observed denialism in downplaying the pandemic’s seriousness or even in the belief in the inefficacy of treatments may result from the lack of government policies to mitigate economic vulnerability during the pandemic. The current study suggests that the guarantee of emergency relief and food security may not only be a key factor for making the lockdown possible but may also favor the population’s adherence to this restrictive measure. Financial issues inherent to health crises must be addressed and communicated appropriately from the outset and not neglected at a later stage as if they were of second importance as this increases the insecurity of those in financial fragility and compromises adherence to measures such as lockdown.

The study’s findings hold significant relevance in the current landscape of global health crises and societal challenges. As the world grapples with ongoing and emerging infectious diseases, understanding the factors that shape individuals’ perceptions and responses to public health measures becomes crucial. The insights gained from this research shed light on the intricate interplay of economic, psychological, and social factors influencing people’s adherence to confinement measures during the COVID-19 pandemic. This knowledge can inform policymakers, public health officials, and researchers in developing more targeted and effective strategies for communication, intervention, and crisis management in the face of similar challenges. Ultimately, the study’s implications extend beyond the immediate context, contributing valuable insights to the broader discourse on pandemic response and public health governance. It is undeniable that weak governance can play a crucial role in understanding the challenges faced during the health crisis. This fragility can impact the population’s trust in institutions and influence their behavior regarding control measures, such as lockdowns.

## Data availability statement

The original contributions presented in the study are included in the article/Supplementary material, further inquiries can be directed to the corresponding author/s.

## Ethics statement

The study was approved by the Research Ethics Committee of the Fernandes Figueira National Institute of Women, Children and Adolescent Health (FIOCRUZ/IFF) with CAAE: 81 45053221.1.0000.5269, through opinion no. 4,640,611, of April 9, 2021, in compliance with Brazilian 82 and international legislation on research involving human beings. The study was conducted in accordance with local legislation and institutional requirements. Participants provided their written informed consent to participate in this study. Written informed consent was obtained from the individual(s) for the publication of any potentially identifiable images or data included in this article.

## Author contributions

KGC: Conceptualization, Data curation, Formal analysis, Investigation, Methodology, Project administration, Resources, Writing – original draft, Writing – review & editing. DCBCM: Writing – original draft, Writing – review & editing, Conceptualization, Data curation, Formal analysis, Investigation, Methodology, Project administration, Resources. MFJ-M: Writing – original draft, Writing – review & editing, Conceptualization, Data curation, Formal analysis, Investigation, Methodology, Project administration, Resources. SCGJ: Writing – original draft, Writing – review & editing, Conceptualization. ATR: Writing – original draft, Writing – review & editing, Conceptualization, Data curation, Formal analysis, Investigation, Methodology, Project administration, Resources. DMA: Conceptualization, Data curation, Formal analysis, Funding acquisition, Investigation, Methodology, Project administration, Resources, Writing – original draft, Writing – review & editing.
