# Supplementary material for: Lockdown during the COVID-19 pandemic: lessons from a polarized scenario in Brazil
Source: Front Psychol. 2024 Apr 10;15:1310594. doi: 10.3389/fpsyg.2024.1310594 (PMC11042250; doi:10.3389/fpsyg.2024.1310594)
Supplement: Supplementary file 1 [file Data_Sheet_1.docx]

Supplementary Table 1 – Beliefs and thoughts on the pandemic and lockdown in the two Clusters identified in the study population.

|  | **Cluster 1** | **Cluster 2** |
| --- | --- | --- |
| **I believe that lockdown will help relieve the overload on health services.**  Agree /totally agree  Neither agree nor disagree  Disagree / totally disagree | 23,417 (99.1%)  163 (0.7%)  52 (0.2%) | 810 (9.8%)  1,203 (14.6%)  6,245 (75.6%) |
| **The financial harm from lockdown can be resolved later. It’s necessary to save lives.**  Agree /totally agree  Neither agree nor disagree  Disagree / totally disagree | 21,682 (91.7%)  1,284 (5.4%)  666 (2.8%) | 350 (4.2%)  843 (10.2%)  7,065 (85.6%) |
| **Lockdown is not possible because Brazil lacks an adequate emergency financial relief policy.**  Agree /totally agree  Neither agree nor disagree  Disagree / totally disagree | 10,872 (46.1%)  3,840 (16.3%)  8,857 (37.6%) | 5,342 (64.8%)  1,204 (14.6%)  1,698 (20.6%) |
| **The economy will not improve unless the**  **pandemic is controlled.**  Agree /totally agree  Neither agree nor disagree  Disagree / totally disagree | 22,791 (96.4%)  397 (1.7%)  444 (1.9%) | 3,351 (40.6%)  1,129 (13.7%)  3,778 (45.7%) |
| **People should not be prevented from coming and going under any circumstances during the pandemic.**  Agree /totally agree  Neither agree nor disagree  Disagree / totally disagree | 761 (3.2%)  1,134 (4.8%)  21,737 (92%) | 6,801 (82.4%)  675 (8.2%)  782 (9.5%) |
| **The harm to the economy from a lockdown would be irreparable.**  Agree /totally agree  Neither agree nor disagree  Disagree / totally disagree | 395 (1.7%)  1,434 (6.1%)  21,803 (92.3%) | 7,439 (90.1%)  587 (7.1%)  232 (2.8%) |
| **We could solve everything with prophylactic treatment (ivermectin, hydroxychloroquine...)**  Agree /totally agree  Neither agree nor disagree  Disagree / totally disagree | 121 (0.5%)  442 (1.9%)  23,069 (97.6%) | 4,531 (54.9%)  2,002 (24.2%)  1,725 (20.9%) |
| **Herd immunity would be the best solution. Everyone that catches COVID would become immune, and the disease would stop spreading.**  Agree /totally agree  Neither agree nor disagree  Disagree / totally disagree | 383 (1.6%)  631 (2.7%)  22,618 (95.7%) | 3,060 (37.1%)  2,387 (28.9%)  2,811 (34%) |
| **I think everyone’s fate is sealed: whoever dies from COVID is bound to die anyway.**  Agree /totally agree  Neither agree nor disagree  Disagree / totally disagree | 271 (1.6%)  1,177 (5%)  22,084 (93.4%) | 1,761 (21.3%)  2,080 (25.2%)  4,417 (53.5%) |
| **I don’t believe the pandemic is as serious as the press claims.**  Agree /totally agree  Neither agree nor disagree  Disagree / totally disagree | 253 (1.1%)  341 (1.4%)  23,038 (97.5%) | 5,494 (66.5%)  1,180 (14.3%)  1,584 (19.2%) |
| **I believe that I have taken effective measures to contain the pandemic.**  Agree /totally agree  Neither agree nor disagree  Disagree / totally disagree | 22,992 (97.4%)  417 (1.8%)  189 (0.8%) | 7,282 (88.3%)  786 (9.5%)  175 (2.1%) |
| **I believe other people have taken effective measures to contain the pandemic.**  Agree /totally agree  Neither agree nor disagree  Disagree / totally disagree | 1,291 (5.5%)  2,636 (11.2%)  19,705 (83.4%) | 2,709 (32.8%)  2,161 (26.2%)  3,388 (41%) |
| **I believe that the government has taken effective measures to contain the pandemic.**  Agree /totally agree  Neither agree nor disagree  Disagree / totally disagree | 863 (3.7%)  1,051 (4.4%)  21,718 (91.9%) | 2,511 (30.4%)  1,999 (24.2%)  3,748 (45.4%) |

Supplementary Table 2 - Fears, feelings, and behaviors toward the pandemic and lockdown in the two Clusters identified in the study population.

| **Fears, feelings, and attitudes** | **Cluster 1** | **Cluster 2** |
| --- | --- | --- |
| **Fear of losing my job or business**  Agree /totally agree  Neither agree nor disagree  Disagree / totally disagree | 6,226 (26.3%)  7,002 (29.6%)  10,404 (44%) | 5,156 (62.4%)  1,618 (19.6%)  1,484 (18%) |
| **Fear that I or my loved ones will get COVID-19**  Agree /totally agree  Neither agree nor disagree  Disagree / totally disagree | 23,262 (98.4%)  191 (0.8%)  179 (0.8%) | 5,796 (70.2%)  1,234 (14.9%)  1,228 (14.9%) |
| **If the lockdown happened, I would feel…**  Very sad/sad  Indifferent  Calm/ very calm | 3,907 (16.5%)  2,170 (9.2%)  17,555 (74.3%) | 7,416 (89.8%)  465 (5.6%)  377 (4.6%) |
| **Lockdown would be a bother for my life.**  Lockdown would not be a bother.  I would not have contact with friends and family.  Lockdown would put my work at risk.  I would not be able to circulate freely in the city.  My mental health would suffer even more.  I would be stuck at home with my children.  I would not be able to enjoy my favorite restaurant or bar.  I would not be able to practice physical exercise.  I would suffer financial losses.  I would have difficulty taking other health treatments | 16,260 (68.8%)  5,171 (21.9%)  1,924 (8.1%)  3,033 (12.7%)  3,269 (13.8%)  1,354 (5.7%)  1,097 (4.6%)  2,692 (11.4%)  2,318 (9.8%)  3,472 (14.7%) | 538 (6.5%)  4,126 (50%)  4,259 (51.6%)  4,371 (52.9%)  5,361 (64.9%)  2,045 (24.8%)  1,239 (15%)  3,883 (47%)  4,369 (52.9%)  4,716 (57.1%) |
| **I usually cope well with social distancing.**  Agree /totally agree  Neither agree nor disagree  Disagree / totally disagree | 16,182 (68.5%)  3,966 (16.8%)  3,484 (14.7%) | 2,592 (31.4%)  1,377 (16.7%)  4,289 (51.9%) |
| **Consequences of lockdown:**  I won’t have money to pay my basic bills.  I’ll get behind on some bills.  It won’t affect my finances.  I’ll be able to profit from this period. | 385 (1.6%)  5,082 (21.5%)  16,856 (71.3%)  70 (0.3%) | 1,713 (20.7%)  2,729 (33.0%)  3,386 (41.0%)  136 (1.6%) |
| **I always wear a face mask in public.**  Agree/totally agree  Neither agree nor disagree  Disagree/ totally disagree | 23,504 (99.5%)  57 (0.2%)  71 (0.3%) | 6,255 (75.7%)  610 (7.4%)  1,393 (16.9%) |
